# The Institutional Worker

**Published:** 1918-08-10

**Authors:** 


THE
### INSTITUTIONAL WORKER
**Being a Special Supplement to "The Hospital"**

### OUR BUREAU OF INFORMATION.

**Rules for Correspondents.**

1. Every letter must be accompanied by the coupon to be cut from the back cover (inside page) of The Hospital, current issue, and must contain the name and address of the correspondent with pseudonym for publication if desired. Replies by post cannot be given save under exceptional circumstances at the Editor's discretion.

2. Letters from Approved Homes in reply to special needs published in the Bureau should state terms and full particulars, and be sent prepaid under cover to the Editor of the Bureau with name written across coupon for identification.

3. Proprietors of Homes which have not yet been entered on the List of Approved Homes, but have spare accommodation likely to suit special needs, are invited to write for an application form for registration. The fee for registration, which includes two announcements of the Home in the Bureau and other privileges, is 10s.

4. All communications to be addressed to the Editor of The Hospital, 28 Southampton Street, Strand, London, W.C. 2, and marked " Bureau of Information."

### INSTITUTION FACTS AND FIGURES.

### The Steward's Department of a Large Hospital.

#### III.—THE PORTER STAFF. REGISTRATION OF PATIENTS.

In a large hospital the control of the porter staff is generally one of the duties of the steward. The organisation of the porters' work will entirely depend on the hospital's way of doing things.

Porters' Duties.

Porters will be required to work in the main entrance, the casualty ward, out-patient department, *post-mortem* department, kitchen, Nurses' Home, etc. These men will have definite work to do from which they should not be called away; they will cope with the work of their particular " point " however varied it may be. For instance, in the Nurses' Home very few nurses may go away on one day, while another day it will be just as much as the Nurses' Home porters can manage to get through the work. It would be little use sending a strange man to assist, for he would not even know his way about the Home.

There should be a certain number of men for emergencies who can leave whatever they are doing at the time to attend to unexpected calls. Clean mattresses may be required to replace dirty ones, patients may have to be taken to the infirmary, male patients to be bathed, and all such calls as these must be dealt with by men who can be taken away without inconveniencing the work. This group of men could fill in their time window-cleaning, taking coals to the wards, changing the dressing and soiled-linen pails, keeping the outside lavatories clean, and in many odd jobs of this sort.

The wages of the porter staff vary according to the position they occupy. The value of all labour has so much increased during war-time, and the conditions of work are so different, that it is almost impossible to set down a hard-and-fast rule as to wages.

Boy labour should be used wherever possible—as, for instance, in cleaning boots, polishing brasses, running messages. It must be borne in mind, however, that whereas a man can do a boy's work when called upon for a short time, boys cannot always do men's labour when the occasion demands. Then, again, a number of boys together are as a rule very noisy, and in a hospital it is very important that the porters and everyone concerned should be as quiet as possible.

Owing to the shortage of men under war conditions it is very difficult for a steward to get the work done efficiently, as he can only use the available labour to the best possible advantage; the work must be done somehow, and generally, with a few exceptions, has to be done by very unskilled people.

Night and Resident Porters.

A certain number of men will be needed at night. The work as a rule is not heavy; but, as in the day-time, anything might occur needing a man to deal with it. Patients are admitted during the night as well as the daytime; patients die in the night as well as in the day, and so on. A certain number of porters should sleep in the hospital, so as to be on the spot for any extra emergencies that may take place. They should be trained to use the fire apparatus in case of necessity. All the porter staff periodically should practise fire drill, not only to know what to do in case of fire, but actually to use the apparatus, for in this way the hose, etc., is always kept in order.

The Registration of Patients.

This work as a rule is carried out in the steward's department. It is important that the register of admissions and discharges should be accurately kept, as inquiries as to the date when a patient was admitted to or discharged from hospital may be made years afterwards. For instance, a man may think he is due for an old-age pension; he does not know when or where he was born, but he does know he was in a hospital when he was quite a child sixty or seventy years ago. The hospital authorities in all probability will be asked if they are able to trace such a name. If the registers have been efficiently kept there should be no difficulty if the man has really been in hospital.

Patients who are dangerously ill must be notified to the steward, so that he can give instructions to the porter on " gate " duty to let the friends visit at any time of the night or day. Arrangements for the patients being transferred to infirmaries are made by the steward. Births and deaths taking place in the hospital have to be communicated to the Registrar. Certificates stating when a patient was admitted and discharged will be given by the steward to patients who need such a statement for any clubs to which they may belong.

The steward's department practically deals, where the patients are concerned, with all public bodies outside the hospital.

☛ Previous articles appeared July 20 and 27.

### AUGUST QUESTIONS.

### 1. War Vacancies.

**Give model "Terms of Appointment" applicable for the substitute of a hospital's administrative official who has been called to the Colours. Provision to be made for the possible return to duty of the official previous to the termination of the war and the filling of the appointment in case of the non-return of the official concerned. Describe fully any experiences in this connection that may prove of value to hospital managers generally.**

### 2. Bedroom Suppers for Nurses Off Duty.

**In those hospitals having no kitchen in the Nurses' Home what arrangements can be made whereby the sisters and nurses who are off duty in the evening may have the comfort of supper in their bedrooms? What method may be adopted in order to avoid the dining-room maid being faced by a more or less depleted pantry when she wishes to lay breakfast? (The difficulty seems to be not so much as regards the food as the crockery.)**

### RULES.

**The following rules must be observed:—**

**1. Contributions must be written on one side of the paper. Brevity and terseness are desirable features. The MSS. must bear the name and address of the sender and be accompanied by coupon to be cut from the back cover (inside page) of the current issue of THE HOSPITAL. A pseudonym must be chosen if the name is not to be published.**

**2. Contributions must be addressed to the Editor of THE HOSPITAL, Institutional Worker Supplement, 28 & 29 Southampton Street, Strand, London, W.C. 2, should reach him before the end of the current month, and be marked in the left-hand corner "Facts and Figures."**

**A minimum payment of Five Shillings will be made for each published answer.**

### QUESTIONS INVITED.

**In connection with our Question Box, we offer every month five shillings each for the two best questions which are sent in for consideration. The questions may be on any institutional subject and concern any department, from the secretary's to the porter's, or the matron's to the domestic staff; the one essential is that they must be practical and deal with points that have a definite relation to hospital or institutional administration, upkeep, finance, or management. Points relating to artificers' work, housekeeping, the laundry, out-patients, and other practical matters will be welcome. It is hoped that thus, when difficult or doubtful points arise in the course of their work, institutional workers will be encouraged to put them in the form of a question and send them to the Editor, THE HOSPITAL Bureau of Information, 28 & 29 Southampton Street, Strand, W.C. 2, marked "Question Box."**

**The best questions will be published in due course and our readers will be given the opportunity of answering them. Thus all institutional workers may be able to cooperate with the view of helping the work and smoothing the difficulties of each other. In short, we want the Question Box of the *Institutional Worker* to be freely used by every worker habitually.**

### ENQUIRIES AND ANSWERS.

### *EMPLOYMENT AND TRAINING.*

### Pupil Housekeeper.

PUPIL housekeepers are received at the London Hospital; Hospital for Consumption, Brompton; and the Hospital for Sick Children, Great Ormond Street, W.C. There is also a School of Housewifery at Northfield, 107-111 Stamford Hill, N.; the Victoria School of Cookery, 4 Onslow Place, South Kensington, S.W.; and St. Martha's College of Housecraft, 4 Chichester Street, St. George's Square, S.W. Write to each particular institution for details of training, etc.—VIOLET.

### Training in Cardiff.

AMONGST the training-schools for hospital nurses in Cardiff are: King Edward VII.'s Hospital; probationers are received for three years' training at the ages of twenty-two to thirty-five; and Union Hospital, Cowbridge Road; suitable applicants are received at any time for three and a half years' training; ages twenty-two to thirty. Write for further particulars to the Matrons of the respective institutions.—MOLLY BAWN.

### *MISCELLANEOUS.*

### Eight-Hour Day for Nurses.

THE difficulties of arranging eight-hour shifts for nurses are not so great as might be anticipated from a casual examination of the system. If the twenty-four hours were divided into three shifts and all nurses were relieved at one time it would take a great many more nurses, and again this plan might lead to some important duty in the wards being overlooked. A hospital matron has suggested that four groups of nurses each working eight hours, the shifts being so arranged that they overlap, is a satisfactory solution of the difficulty. First shift: Duty after breakfast at 7.15; dinner 12 to 12.30; off duty at 3.45. Second shift: Duty 8.30 A.M.; dinner at 12.30; off duty until 3.30 P.M.; return to duty until 7.30 P.M. or 8 if necessary. Third shift: Commence duty 3 P.M.; supper 5.30; off duty 11.30 P.M. Fourth shift: Commence duty 11.30 P.M., after a dinner served at 11 P.M.; off duty 7.30 A.M.—MATRON.

### Paper Bandages.

THE shortage of cotton due to war conditions has led to the substitution of other material for the purpose of surgical dressings. One of the most recent introductions is the crêpe-paper bandage made by the Dennison Manufacturing Co., of Kingsway, W.C. 2. This bandage is wonderfully strong and elastic, and in many respects more serviceable than a cheap fabric bandage. The prices range from 184*s*. 6*d*. per thousand 1-in. bandage to 512*s*. 6*d*. per thousand 4-in. bandages. —AMY.

### Substitute for Absorbent Cotton

AMONG other substitutes for absorbent cotton-wool is cotton-cellulose, which is a very fair substitute for the absorbent wool which in the days of plenty was considered indispensable. It is chemically pure and is sterilised during the process of manufacture by being subjected to a temperature of 350°F. for a period of ten hours. The material is highly absorbent, and the price is lower than that of cotton-wool. It is supplied by Messrs. Allen and Hanburys, Ltd., 48 Wigmore Street, Cavendish Square, London, W.—ECONOMIST.

### Cracks in Concrete Floors.

No successful method has apparently been devised for preventing the cracking of concrete floors. It is due to variations in temperature and consequent expansion and contraction or to slight settlements of portions of the building. It is not advisable in the case of a new building to do more than fill up the crack. When settlement has ceased the cracks may be cut out and stopped with concrete, and the surface matched with terrazzo or such other composition as has been used. It does not follow, however, that the defect will not recur.—Y. B. P.

### Waste Paper.

THE need for conserving waste material is becoming increasingly apparent, and it is surprising to note the high prices which can be obtained for refuse which at one time would have been regarded as valueless. £12 per ton for good office waste paper can be now readily obtained; at one time £2 2s. a ton was considered a fair price. For better grades of paper, sorted, a higher price even than this can be obtained.

### Grease Trap.

THE trouble with your kitchen sink is that you have no grease trap, and until you have this defect remedied the annoyance will continue to recur. An excellent type of submerged grease extractor is made by the Canadian-American Machinery Company, of 8 Bouverie Street, Fleet Street, London, E.C. 4. It is well to remember that grease is very valuable in these days, and should not be allowed to run to waste.—M. W.

### Old Tins.

THE demands by the Munitions Department for tinned plate and the consequent shortage of this material for domestic purposes has developed a market for old tins, for which prices ranging up to £20 a ton can now be obtained. It is important to know, however, that this price is only paid if the tins are cut and partly flattened and free from rust. A special machine has been devised for cutting away the solder seam, which may also be conserved for reclaiming the solder. A large quantity of this can be stored in a small space when flattened out in this way.—JENNIE.

